# Microspheres Formation in a Glass–Metal Hybrid Fiber System: Application in Optical Microwires

**DOI:** 10.3390/ma12121969

**Published:** 2019-06-19

**Authors:** Afroditi Petropoulou, Dimitris Drikakis, Christos Riziotis

**Affiliations:** 1 Theoretical and Physical Chemistry Institute, National Hellenic Research Foundation, 116 35 Athens, Greece; apetropoulou@eie.gr; 2 Department of Informatics and Telecommunications, University of Peloponnese, 22100 Tripolis, Greece; 3 School of Sciences and Engineering, University of Nicosia, Nicosia CY 2417, Cyprus; drikakis.d@unic.ac.cy

**Keywords:** micro wires, fibers, metals, glass, instabilities, microfluidics, optics, simulations

## Abstract

Multicomponent optical fibers with incorporated metals are promising photonic platforms for engineering of tailored plasmonic structures by laser micromachining or thermal processing. It has been observed that during thermal processing microfluidic phenomena lead to the formation of embedded micro- and nanostructures and spheres, thus triggering the technological motivation for their theoretical investigation, especially in the practical case of noble metal/glass composites that have not yet been investigated. Implemented microwires of gold core and glass cladding, recently studied experimentally, are considered as a reference validation platform. The Plateau-Rayleigh instability in such hybrid fibers is theoretically investigated by inducing surface tension perturbations and by comparing them to the Tomotika instability theory. The continuous-core breakup time was calculated via Finite Element Method (FEM) simulations for different temperatures and was found to be considerably higher to Tomotika’s model, while the final sphere diameter is a linear function of the initial core radius. Different sinusoidal perturbation parameters were considered, showing significant impact in the characteristics of formed spherical features. The theoretical results were in close agreement with previous experimental observations expected to assist in the understanding of the processes involved, providing insight into the engineering of fibers, both in the initial drawing process and post processing.

## 1. Introduction

The need for tailoring the properties of optical fibers has resulted in the design and fabrication of hybrid multicomponent material fiber structures. A special class of composite optical platforms consists of a combination of a supporting glass structure and noble metals that could give rise to plasmonic effects for a class of nanofocusing photonic applications, such as microscopy, data storage [1], and sensing [2,3], as well as continuum light generation [4]. 

Although the drawing of fibers containing base metals has already been investigated [5], the study of fibers containing noble metals is of intense interest for plasmonic devices. The manufacturing and post processing [6,7] of such fibers though is quite challenging due to the combination of materials with different mechanical and physicochemical properties. At high temperature processing conditions, a number of instabilities induce various microfluidic phenomena leading in turn to formation of various embedded microstructures. The understanding of those phenomena is crucial, as it would help either to avoid such structures or help in their tailored fabrication, consequently setting the motivation for their theoretical investigation. The choice of materials with compatible properties, such as melting and working points, is crucial in order to successfully draw uniform and low loss fibers. For the fabrication of fibers with uniform diameters and continuous metallic cores, key parameters, such as feed rate of the metal, temperature, and pull rate, should be optimised during the drawing process [5,8]. In the high temperature conditions needed for the fabrication of such multi-material systems, the breakup of the core is commonly observed, and the consequent formation of spheres, and thus the temperature profile, must be carefully controlled. This breakup is attributed to the Plateau-Rayleigh instability, which grows in the low viscosity regime. Furthermore, as the diameter of the metal decreases, the instability increases, limiting the reduction of the fibers’ diameters in the nanoscale, where fragmentation of the metal can occur, even at temperatures lower than the bulk melting temperature [9,10]. 

Although the breakup of the core needs to be avoided during the fabrication and post-processing of hybrid optical fibers, the study of the formation of an array of spheres with customised diameters could be of great value for many applications, such as the development of integrated optical microresonators [11]. Recent studies of capillary instabilities [12,13,14] have paved the way towards this direction. Chains of dielectric [15,16,17] and Cu [18] nanoparticles have already been demonstrated, showing high control over both size and spacing of the nanoparticles. The formation of noble metal micro-nano spheres in an all-fiber plasmonic device, such as the one proposed here, would offer mechanical robustness, ease of light coupling, as well as the possibility of its integration into standard optical fibers, enabling the remote use of the device.

In the present study, the investigated platform consists of a gold core with a surrounding borosilicate glass cladding with typical diameters of 4 μm and 40 μm, respectively. It was chosen to organise this theoretical study around a specific composite fiber-optic platform, as it served as a motivation engineering case that could also allow comparison of the findings with recently obtained experimental results [6].

**Figure 1:**
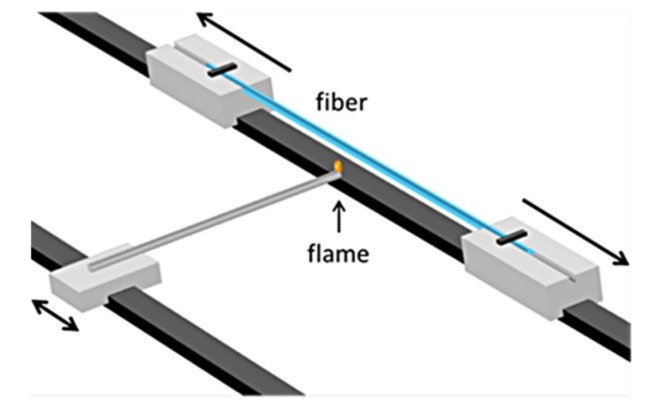
Schematic representation of a typical fiber tapering rig.

The theoretical investigation of the dynamic phenomena during the processing of composite metal/glass structures and specifically fibers would allow the understanding of underlying fundamental phenomena and will enable the optimization of both the fiber drawing manufacturing process, as well as their post processing. A special case of post processing, also studied here, is the tapering of the fibers for the implementation of nanoscale plasmon tips for light nanofocusing. The heating and stretching method [19] is a widely used technique for the fabrication of optical fiber tapers. The two fiber ends are placed at two elongation stages, inside optimum sized grooves. A third stage holding the heating element moves across the fiber axis over several millimetres to heat the fiber (Figure 1). A flame produced by a butane-oxygen mixture can be used as the heating element. The temperature profile can be adjusted by changing the flow rates of the gases, as well as the distance between the fiber and the flame. Appropriate conditions, such as the velocity and temperature of the heating element, as well as the pull rate of the fiber, can lead to the fabrication of smooth plasmon tips with the desirable geometrical characteristics [6]. In this direction, microfluidic simulations of the tapering process were performed for the determination of the appropriate conditions that would allow the development of plasmonic tips.

The above discussion supports the need for further understanding of the dynamic phenomena that occur during the thermal processing of such hybrid microfibers. Therefore, in this work a study is conducted for the first time, to the best of our knowledge, on the microfluidic phenomena in a microfiber platform of silicate glass and a noble metal core, with significant anticipated applications. Microfluidics simulations based on Finite Element Method (FEM) were performed and compared with the Tomotika’s linear stability analysis [20]. The study of the instabilities induced by the temperature profile, such as perturbation wavelength and width in the case of static heating, can be used as a guide for the fabrication of fibers with desirable characteristics. It is shown that although the diameter of the primary spheres does not depend on the temperature, but only on the initial core radius, small variations of the spheres’ sizes and of the number of the secondary spheres can result from different perturbation wavelengths. Furthermore, the time that a part of the fiber remains under the influence of the perturbation is an important parameter for the stability of the metal core. For short time scales the core can remain intact while for longer processing time breakup occurs [21]. The breakup time calculated by the simulations was found to be longer than the time estimated from the Tomotika model due to Tomotika’s assumption of an unbound surrounding medium. This is in accordance with our previous experimental results [6], where the breakup of the core was prevented during the post-processing of the microfibers towards the fabrication of fiber tips, as described above. For the short time scales estimated by the Tomotika model, this would not be feasible with our experimental setup (Figure 1), due to the limited velocity of the flame. Microfluidic simulations of the tapering process were also performed, showing that for low temperatures close to the melting point of gold, smooth fiber tips can be developed. 

## 2. Materials and Methods

**Figure 2:**
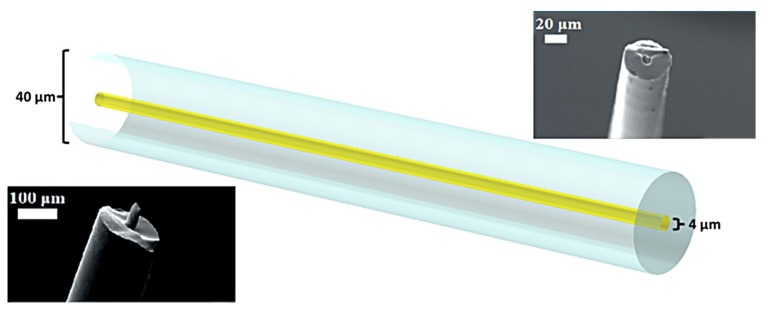
Schematic of a 4 μm gold core/40 μm borosilicate glass cladding fiber. The insets present characteristic SEM images of actual microwires of different diameters. Adapted with permission from [6]. Copyright 2018 American Chemical Society.

For this study we consider a fiber with a gold core surrounded by a large borosilicate glass cladding (Figure 2). The borosilicate glass was chosen due to its temperature compatibility with the gold. The working range of borosilicate glass is between 825 °C and 1260 °C, which overlaps with the melting point of gold (1065 °C). Both the core and the Schott Duran borosilicate glass cladding viscosities, as a function of temperature, are taken into account, with μ_gold_(T) [22] and μ_glass_(T) (obtained from the technical data sheet by fitting a power law curve to the given viscosities), respectively. The interfacial surface tension, s(T) is considered to be dominated by the surface tension of gold.

Instability analysis based on Tomotika’s stability theory [20] is performed for temperatures ≥1065 °C, for which the gold core is in its liquid form. Tomotika’s theory is strictly applicable only when the relative velocity of the jet and surrounding fluid are very low. Hence, for the static case studied here, we can use the linear stability analysis of Tomotika, and we can calculate the breakup time τ_Β_ [21], defined as:

(1) $$\tau_{Β}\left(Τ\right)=\frac{1}{\mathrm{in}},$$

where

(2) $$\mathrm{in}=\frac{s}{2{r\mu }_{\mathrm{glass}}}\left(1-x^{2}\right)\Phi \left(x\right),$$

with r being the core radius, x = 2πr/λ, and Φ the function (39) in general cases of the Tomotika model [20].

For the Finite Element Method (FEM) simulations, FEM-based COMSOL Multiphysics Modeling software (Version 4.4, COMSOL Inc.) was employed to solve the Navier-Stokes equations that describe the velocity field and pressure of the liquid. The level set method was chosen, in which the fluid–fluid interface is represented as the 0.5 contour of the level set function. The level set method was chosen over the moving mesh method, since the topology of the core after its breakup is of interest. Recent developments on the improvement of the level set method can be found in previous studies [23,24,25]. No-slip walls were set as boundary conditions. Periodic boundary conditions were also used to describe an infinitely long fiber. All the simulations are run with extra fine mesh and for times up to 0.3 s, since for higher times the model does not converge. Furthermore, since the length scales considered here are well below the capillary length, λ_c_ = 2.55 mm, the gravitational effects are neglected. This assumption was supported also by the fact that in the experimental results no signs of gravitational effects were obvious, since the fibers were symmetric with respect to the fiber axis after the formation of the spheres.

## 3. Results and Discussion

The theoretical investigation is divided in two distinct approaches—the instability analysis based on the Tomotika model and the FEM study. The theoretical results are also compared with experimental findings, drawing conclusions on the validity of the model and gaining physical insight into the microspheres formation process.

### 3.1. Instability Analysis of Heated Microwires

**Figure 3:**
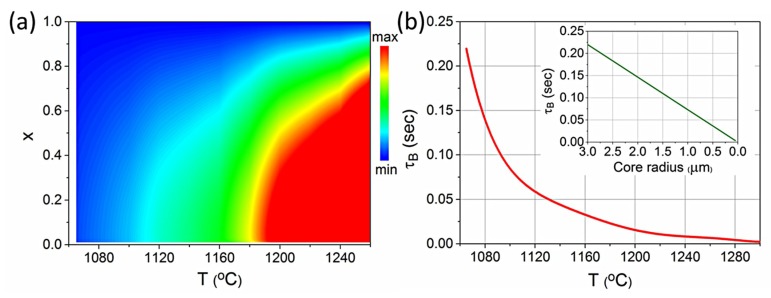
(**a**) Instability rate (1/τ_B_) as a function of T and x for core radius 3 μm. (**b**) Breakup time τ_B_ as a function of temperature. Inset picture: Breakup time τ_B_ as a function of core radius at 1065 °C.

Using the equations of the general case of the Tomotika model, we can calculate τ_B_ as a function of the temperature (Figure 3a). In the case studied here, the ratio of the core to cladding viscosity tends to be zero ($\frac{\mu_{\mathrm{gold}}}{\mu_{\mathrm{glass}}}\to 0$), which according to the Tomotika model leads to a maximum instability for x→0, or accordingly for perturbation wavelength λ to be much larger than the radius of the core (which typically is ~3 μm). As the temperature decreases the viscosity of the surrounding glass (μ_glass_) increases exponentially, leading to a stable jet due to the rapid increase of τ_B_, according to Equation (1).

When such fibers are tapered down to small core radii, the time that they remain in the heating zone is crucial in order to prevent the breakup of the core. In Figure 3b, τ_B_, corresponding to the maximum instability, i.e., to the x that maximizes Equation (2), is presented as a function of the temperature. For 1065 °C, which is the melting point of gold, τ_B_ is ~0.22 s for a 3 μm core radius. This value linearly decreases for smaller core radii, as shown in the inset diagram of Figure 3b. Hence, a static heating zone would not be appropriate for the tapering of the fibers, as it would lead to the rapid collapse of the core. On the contrary, a moving heating source, such as a moving flame, can be used to successfully taper hybrid fibers. 

The flame’s temperature profile and distance from the fiber must be adjusted to match the desirable temperature of 1065 °C, as for higher temperatures the instability of the core will increase. The most crucial parameter though is the flame’s velocity. Since τ_B_ is ~0.22 s, a high flame velocity is needed. Also, as the fiber is tapered down, the core radius decreases and becomes more unstable. Hence, the flame’s velocity must be adjusted in order to be fast enough to prevent the breakup of the core and slow enough to permit the melting of the metal. Furthermore, due to the system’s high instability, a fast tapering process would be preferable. Thus, the pulling velocity of the fiber should be high enough to reduce the tapering time.

### 3.2. Simulation Studies

#### 3.2.1. Effect of Temperature

In order to investigate the instability of a gold cylindrical rod with a 3 μm radius surrounded by a borosilicate glass cladding, we assume a surface tension perturbation of the following form:

(3) $$s=s_{0}\left(1-\left(w*p\left(t\right)*\cos\left(\frac{2*\pi *z}{\lambda }\right)\right)\right),$$

where s_0_ is the surface tension corresponding to the desirable temperature, w is the width, and λ is the wavelength of the perturbation, respectively, and p(t) is a piecewise function that controls the time that the fiber remains under the influence of the perturbation. Note that perturbations are not possible to be measured experimentally, thus due to the stochastic nature of the problem, both experiments and simulations encompass a degree of uncertainty, which should be borne in mind when comparisons are performed. 

**Figure 4:**
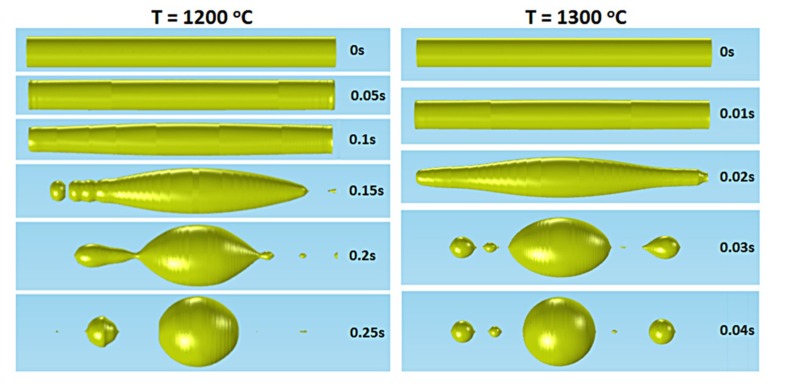
Different stages of the Plateau-Rayleigh instability during static heating for different temperatures (1200 °C and 1300 °C) of a gold cylindrical rod by inserting a surface tension perturbation.

Figure 4 shows the modeled region of the fiber at different time steps at 1200 °C and at 1300 °C. The parameters used are w = 0.008, λ = 200 μm and

(4) $$p\left(t\right)=\;[\begin{array}{r} 10^{-2t}\;t<0.2\;s\\ 0\;\;t>0.2\;s \end{array}\;,$$

The core at T = 1300 °C breaks ~10 times faster than T = 1200 °C, as expected due to the lower viscosity of gold. Furthermore, the size of the primary sphere does not seem to depend on the temperature, but only on the initial core radius. Simulations were also performed for different temperatures: 1065 °C, 1100 °C, 1150 °C, 1180 °C, 1200 °C, 1250 °C, and 1300 °C. For temperatures up to 1180 °C and for the simulation time of 0.3 s, the final sphere is not formed but still has an ovular shape, and hence the diameter cannot be measured. For temperatures of 1200 °C, 1250 °C, and 1300 °C, the size of the primary sphere diameter is consistently around 14.3 μm, as indicatively shown below in Figure 4. 

**Figure 5:**
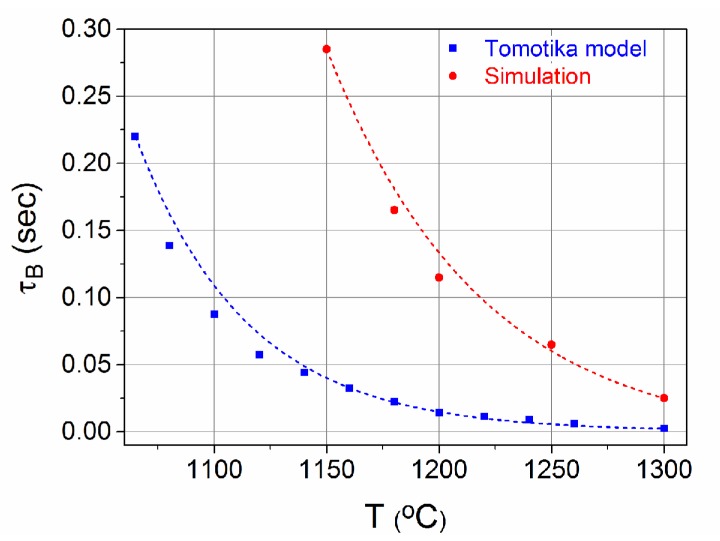
Breakup time as a function of temperature calculated using simulations (red) and the Tomotika model (blue).

Figure 5 shows τ_Β_ as a function of the temperature. For temperatures lower than ~1150 °C, the core remains intact for times up to 0.3 s, according to the simulations. The calculated τ_Β_ is 10 times higher than the one calculated from the Tomotika model. This is attributed to the fact that the Tomotika model assumes an infinite unbound surrounding fluid. However, for the simulations a limited surrounding fluid is considered with an external radius corresponding to real microwires. As it has been shown by Liu et al. [26], the instability grows as the thickness of the surrounding medium increases, which justifies the discrepancies in τ_Β_ between the Tomotika model and simulations. Therefore, the comparisons should be considered from the qualitative viewpoint, only bearing in mind that the physics of the problem is highly non-linear, thus cannot be fully captured by Tomotika’s model.

#### 3.2.2. Effect of Core Radius

**Figure 6:**
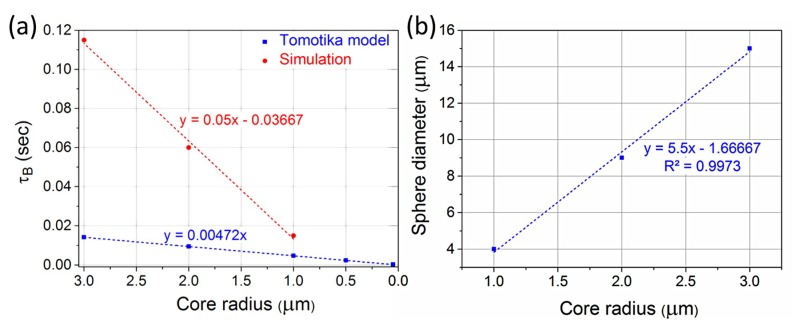
(**a**) Breakup time as a function of the initial core radius at T = 1200 °C calculated using simulations (red) and the Tomotika model (blue). (**b**) Sphere diameter as a function of the core radius at T = 1200 °C.

Simulations were also performed for the calculation of τ_Β_ for different initial core radii. The temperature for the simulations was 1200 °C, and as seen from Figure 6a, τ_Β_ is a linear function of the core radius, which is in agreement with what is expected from the Tomotika model. 

Furthermore, the diameter of the formed spheres is also a linear function of the core radius, as shown in Figure 6b. The temperature for the simulations was 1200 °C, although the final sphere diameter does not seem to depend (at least at a considerable or measurable degree) on the temperature, as has been indicated by the discussion above, in Figure 4, where for temperatures of 1200 °C, 1250 °C, and 1300 °C, the size of the primary sphere diameter is consistently around 14.3 μm. Although this conclusion cannot be fully validated at this stage it can be intuitively explained as follows. The sphere diameters do not depend on the intermediate transient effects, which differ as functions of temperature, but on at the final equilibrium condition set by the structure’s geometrical characteristics.

**Figure 7:**
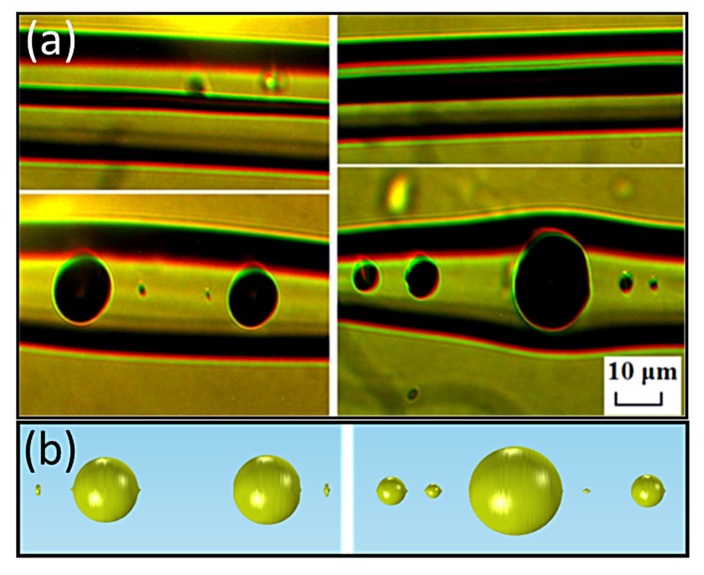
(**a**) Microscope images of formed spheres from fibers with initial core radii of 2 μm (left) and 3 μm (right). Reprinted with permission from [6]. Copyright 2018 American Chemical Society. (**b**) The resulting topology of 2 μm (left) and a 3 μm (right) gold core radii.

Figure 7 shows microscope images of spheres formed from fibers with different initial core radii. Specifically, the spheres were obtained when the temperature and the velocity of the flame were 1200 °C and 2 mm/s, respectively. The diameters of the spheres are approximately 12 μm and 17 μm for 2 μm and 3 μm initial core radii, respectively, a result close to the simulations, where the corresponding diameters were ~9 μm and ~14.3 μm (Figure 6b). The distance between the spheres is ~37 μm, while for the simulated ones it is 22.5 μm.

#### 3.2.3. Effect of the Width and Wavelength of Perturbations

**Figure 8:**
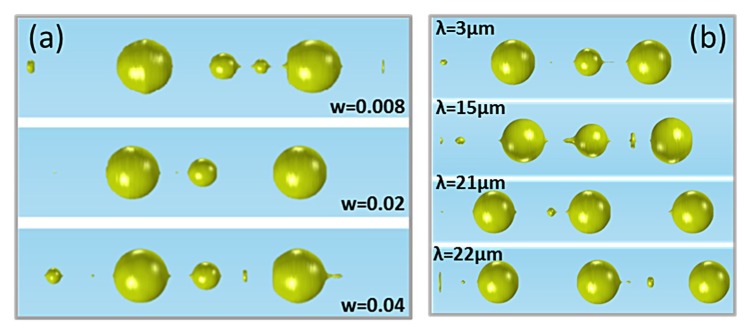
(**a**) The resulting topology of a 2 μm radius gold core for different widths of the surface tension. (**b**) The resulting topology of a 2 μm radius gold core for different wavelengths of the surface tension.

Simulations for surface tension perturbations with different widths and wavelengths were also performed to investigate the differences between the formed microspheres. The temperature is considered constant at 1200 °C. The surface tension perturbation has the following form:

(5) $$s=s_{0}\left(1-\left(w*\cos\left(\frac{2*\pi *z}{\lambda }\right)\right)\right),$$

where s_0_ is the surface tension corresponding to the desirable temperature, w is the width, and λ is the wavelength of the perturbation. Figure 8a,b shows the resulting topology for different widths and wavelengths of the surface tension, respectively. Although the distance and size of the primary spheres remain the same, the number and sizes of the formed satellite spheres differ significantly. Furthermore, the calculated τ_B_ is the same for different widths. For the simulations of Figure 8a, the wavelength was 5 μm and the widths were 0.008, 0.02, and 0.04. For the simulations of Figure 8b, the width was 0.008 and the wavelengths were 3, 15, 21 and 22 μm. 

**Figure 9:**
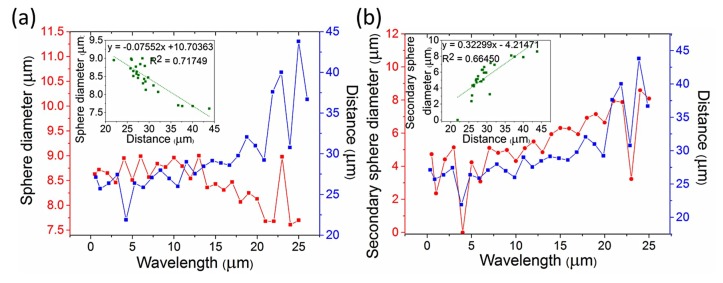
(**a**) Diameter of the primary spheres and distance between them as a function of the perturbation wavelength. (**b**) Diameter of the secondary sphere and distance between the primary spheres as a function of the perturbation wavelength.

The diameters of the primary and secondary formed spheres as a function of the wavelength are shown in Figure 9a,b, respectively. Only perturbation wavelengths up to 25 μm are presented due to the computationally limited simulating fiber length. The distance between the primary spheres is related to the diameters of both the primary and secondary spheres. As primary spheres, only the two outer spheres of Figure 8b are considered for all the calculations, even when the secondary (middle) sphere reaches the size of the primary. For larger secondary spheres the size of the primary spheres decreases due to mass conservation. Furthermore, a large secondary sphere between two primary spheres means that the breakup points have larger distances between them, leading to larger distances between the two primary spheres. This explains the anti-correlation between the diameter of the primary spheres and their distance (with Pearson and Spearman correlation coefficients −0.84705 and −0.71876, respectively) and the correlation between the diameter of the secondary spheres and the distance between the primary spheres (with Pearson and Spearman correlation coefficients −0.81517 and −0.85451, respectively), as shown in the inset plots of Figure 9a,b. The values of the slopes for the inset plots of Figure 9a,b are −0.07552 and −0.32299, respectively. With corresponding standard errors (0.00967 and 0.04685, respectively) being less than 20% of the slope values, we can consider the correlation in both plots linear. 

Although the primary sphere diameter and the distance between the primary spheres seem to have a linear anti-correlation, this can only be valid for a rather short range of diameters and distances. The minimum diameter of a sphere is $\sqrt[{3}]{\frac{3\pi }{2}}D$ (corresponding to a minimum distance of πD), where D is the initial core diameter. For a 4 μm core, the corresponding minimum sphere diameter and minimum distance are D_min_ = 6.3 μm and d_min_ = 12.6 μm, respectively, in the case where only primary spheres exist. When secondary spheres are present, the distance between two primary spheres increase with the wavelength until the size of the secondary sphere reaches the size of the primary, and hence it is also considered as primary (Figure 8b). This result is obvious in Figure 9b, where the diameters of the secondary spheres for long wavelengths are ~8 μm, comparable to the diameters of the primary spheres shown in Figure 9a. The perturbation wavelength at which three equally sized spheres (D ≈ 8 μm) are formed for an initial 4 μm core is λ ≈ 21 μm and the distance between the spheres is d ≈ 19 μm, slightly larger than the theoretical D_min_ and d_min_, probably due to the existence of smaller satellite spheres. This result is also very close to the experimental distance between the spheres, as shown above, where d ≈ 37 μm (Figure 7a). As shown in Section 3.2.2, the diameter of the primary sphere depends on the initial core radius and is the same for different temperatures. In Figure 9 only small variations of the primary sphere diameters are seen, with an average diameter of 8.84 μm, which is the expected diameter for a 2 μm core radius, as shown in Figure 6b, where a wavelength of 200 μm was considered, leading to a large distance between the spheres. Hence, for large distances between the primary spheres the primary sphere diameter will remain ~9 μm for a 2 μm initial core radius but the number of the secondary spheres will increase due to mass conservation.

The formation of secondary spheres is in agreement with previously published works, where satellite droplets are observed [11,21]. Furthermore, we have control over the primary sphere diameter, since, as discussed above, it is only related to the initial core radius. The control of the sphere’s diameter over a small range has been previously shown [15], where silica-clad silicon-core fiber with a diameter of 340 nm was continuously fed into a flame, defining an axial thermal gradient; the continuous formation of spheres whose size is controlled by the feed speed was also demonstrated. Due to the limited simulated length though we cannot come to a conclusion concerning the distance between the primary spheres, since only 1 or 2 spheres are formed in this limited segment.

#### 3.2.4. Simulation of the Tapering Process

The fibers’ tapering process, as described in the introduction and illustrated in Figure 1, was also simulated in order to study the conditions of smooth adiabatic tapering by avoiding the formation of spheres. For the purposes of the simulation a radial velocity was added to the outer glass boundary using deformed geometry. The velocity was set to be:

(6) $$v_{r}=-0.5\left(z+30\right)\;(\mu m/s),$$

with z being the axis along the fiber ranging from −30 μm to 30 μm. 

**Figure 10:**
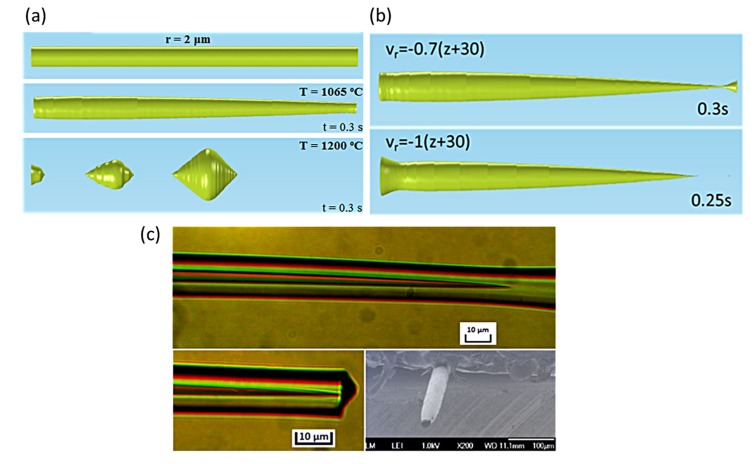
(**a**) Simulation of the tapering process of a 2 μm core radius microfiber at 1065 °C and 1200 °C with v_r_ = −0.5 (z + 30) (μm/s), showing the core’s evolution after 0.3 s. (**b**) Simulation of the tapering process of a 2 μm core radius microfiber at 1065 °C for higher radial velocities. (**c**) Top: Optical microscope images of a fabricated fiber tip before cleaving. Bottom: Optical microscope (left) and SEM (right) characteristic images of a fabricated fiber tip after the cleaving process (Reprinted with permission from [6]. Copyright 2018 American Chemical Society).

Two different temperatures, 1065 °C and 1200 °C, were investigated. The simulation time was 0.3 s. The results are shown in Figure 10a. At 1065 °C a smooth taper is obtained, while at 1200 °C the metal core breaks into spheres. Even though the velocity is rather high compared to the experimental procedure, it was chosen in order to test the behavior of the core when its diameter reduces drastically. Even higher velocities result in the formation of smooth tapers for T = 1065 °C (Figure 10b). For lower velocities the simulation should run for longer time scales in order to reach the same diameters, which are limited due to convergence issues. However, lower and higher velocities were also simulated, giving the same results, i.e., smooth tapers at 1065 °C and core breakup at 1200 °C.

The performed simulations and the study of instabilities in the heated microwires provided very useful and intuitive guidelines for the appropriate thermal treatment in the tapering process. Indeed, when the lowest possible temperature (~1065 °C) combined with high flame velocity (6 mm/s; “fast and cold method”) were employed in the experimental tapering process, which is schematically represented in Figure 1, smooth adiabatic tips were successfully fabricated (Figure 10c) [6].

## 4. Conclusions

Motivated by recent experimental results, we investigated the process of microsphere formation in a glass/metal hybrid microfiber. FEM simulations were performed in order to investigate, for the first time, microfluidic phenomena in hybrid metal/glass microfibers, by adopting a specific physically implemented microfiber platform consisting of gold core microwires surrounded by glass borosilicate cladding. Simulations revealed the conditions for the formation of various discontinuous features, such as primary and secondary microspheres after the breakup of the solid gold core due to Plateau-Rayleigh instability. The results differ from the Tomotika instability theory, which assumes an unbound surrounding fluid. Different gold core diameters were considered, showing that there is a linear dependence of both the breakup time and final primary sphere diameter to the initial core size. By changing the width and wavelength of the surface tension perturbation, significant differences of the final formed satellite spheres were observed, providing useful insight in the process. The theoretical results were in good agreement with experimental findings [6], thus providing an intuitive view of the underlying process and useful guidelines for controllable fabrication of in-fiber features, such as spheres or tapered tips for photonic applications. It is suggested that future work should also take into account multiscale phenomena across micro- and nanoscales using hybrid molecular-continuum models [27]. This will lead to a better understanding of the effects of perturbations on the instability formation on microscale.
